# Perovskite Oxide–Based Materials for Photocatalytic and Photoelectrocatalytic Treatment of Water

**DOI:** 10.3389/fchem.2021.634630

**Published:** 2021-04-15

**Authors:** Oluchi V. Nkwachukwu, Omotayo A. Arotiba

**Affiliations:** ^1^ Department of Chemical Sciences, University of Johannesburg, Johannesburg, South Africa; ^2^ Centre for Nanomaterials Science Research, University of Johannesburg, Johannesburg, South Africa

**Keywords:** perovskites, water treatment, photocatalysis, photoelectrocatalysis, degradation

## Abstract

Meeting the global challenge of water availability necessitates diversification from traditional water treatment methods to other complementary methods, such as photocatalysis and photoelectrocatalysis (PEC), for a more robust solution. Materials play very important roles in the development of these newer methods. Thus, the quest and applications of a myriad of materials are ongoing areas of water research. Perovskite and perovskite-related materials, which have been largely explored in the energy sectors, are potential materials in water treatment technologies. In this review, attention is paid to the recent progress in the application of perovskite materials in photocatalytic and photoelectrocatalytic degradation of organic pollutants in water. Water treatment applications of lanthanum, ferrite, titanate, and tantalum (and others)-based perovskites are discussed. The chemical nature and different synthetic routes of perovskites or perovskite composites are presented as fundamental to applications.

## Introduction

A perovskite by definition is a material with the same crystal structure as CaTiO_3_, BaTiO_3_, CaSiO_3_, or SrTiO_3_. Perovskite mineral was discovered in the Ural Mountains, Russia, in 1839. A German mineralogist and crystallographer named Gustav Rose received the samples from Alexander Kämmerer—a Russian mineralogist. Rose did a lot of work on the determination of the properties of perovskite, and taking that into account, he named this mineral after Lev Perovski, who was a Russian politician and mineralogist (Park et al., 2016; Katz, 2020; Etgar, 2016). Typically, perovskites are binary metal oxides with a general formula ABO_3_, where A cation can be lanthanide, alkaline, or alkaline earth cation, and B cation is a metallic element with 3-, 4-, or 5-day configuration (Arandiyan et al., 2018). Perovskite and perovskite-related materials have emerged as an important new class of materials due to their fascinating physicochemical properties such as thermal stability, electron mobility, and redox behavior (Zhu et al., 2015; Arandiyan et al., 2018), and their versatile applications in catalysis, water splitting, solar cells, optical devices, and superconductors (Khalesi et al., 2008a; Khalesi et al., 2008b).

**FIGURE 1:**
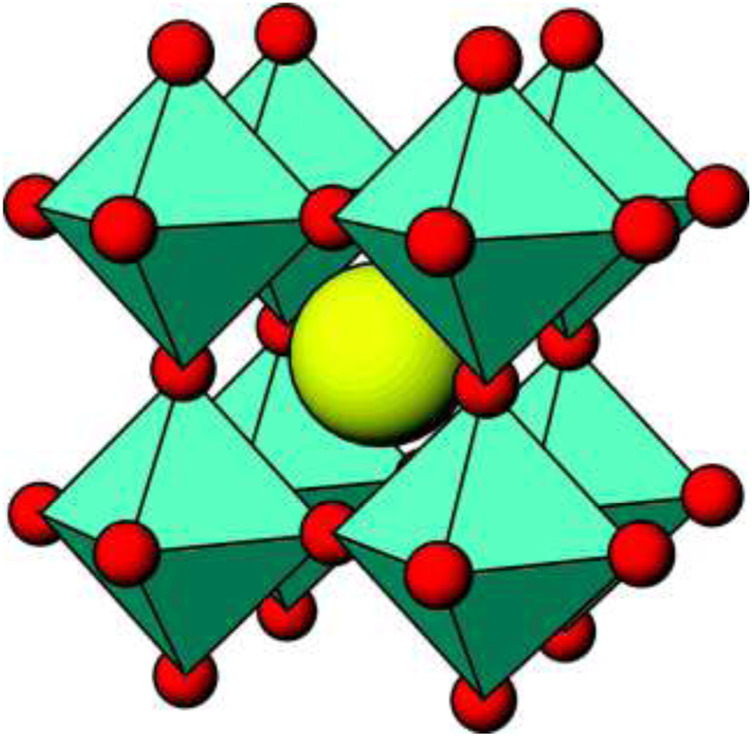
Ideal cubic perovskite structure for ABO3 (cyan, BO_6_ units; yellow, A atoms; Kubacka et al. (2012).

An ideal perovskite structure has an ABO_3_ stoichiometry and a cubic crystal structure (Figure 1). The cubic cell is composed of a three-dimensional framework of corner-sharing BO_6_ octahedra. The B-site cation is a transition metal element (Kubacka et al., 2012). The A-site cation occupies the 12 coordinate position formed by the BO_6_ network and often consists of an alkaline earth metal element or a rare earth element. With comparison to the ideal cubic perovskite ABO_3_, perovskite-related structures arise from the loss of one or more of the symmetry operator in the cubic structure and exhibit lattice distortion to varying degrees, thereby resulting in a nonideal structure of the crystal phases such as orthogonal, rhombohedral, tetragonal, monoclinic, and triclinic phases. Although primitive cubic is the idealized structure, the differences in radii between both cations can in fact distort the structure. This normally involves tilting of the BO_6_ units (octahedral tilting; Kong et al., 2019). This distortion is created in the crystalline structure as perovskites adopt a wide range of different composition by partially substituting either A or B cation of the same or different valences, resulting in a general formula A1-xAx’B1-yB’yO3±δ, where “+” denotes oxygen excess and “−” denotes oxygen deficiency. It is important to note that both A and B or either offers great flexibility with regard to tailoring and tuning of the physicochemical properties (Zhang et al., 1990; Zhu et al., 2015; Torregrosa-Rivero et al., 2017; Arandiyan et al., 2018). For example, Gade et al. (2018) studied the photocatalytic efficiency in ALaTi_2_O_6_, where A is Na, Ag, or Cu. This new class of material efficiently and effectively mineralized the Congo red dye, 4-chlorophenol, and 4-4′-bis (2 sulfostyryl) biphenyl used as model pollutants (Gade et al., 2018). The stability of the perovskite is summarized by the Goldschmidt tolerance factor, *t* = (rA + rO)/ $\;\sqrt{2}$ (rB + rO), where rA, r_B_, and r_O_ are the, respective, radii of A, B, and oxygen ions. For the perovskite structure, the Goldschmidt tolerance factor lies between 0.76 and 1.13 (Zhu A. et al., 2014; Acharya et al., 2015; Kumar et al., 2019).

The diverse composition of emerging pollutants in water suggests that no single method of water treatment can be termed universal. This necessitates the exploration of newer water treatment methods and the tailoring of these methods to certain segments of wastewater. In response to this water treatment challenge, advanced oxidation processes (AOPs) have been developed as an effective technology to remove persistent organic pollutants from wastewater. AOPs are based on *in situ* generation of radicals that nonselectively react with most organics and are able to degrade highly recalcitrant compounds (Brillas and Martínez-Huitle, 2015; Garcia-Segura and Brillas, 2017; Peleyeju and Arotiba, 2018; Umukoro et al., 2018). This review is in addition to the existing body of knowledge around AOPs for water treatment. It is however distinct as it focuses on the application of perovskites in advanced oxidation processes related to water treatment. The so-called first-generation photocatalysts such as TiO_2_, SnO, and ZnO have the limitations of wide bandgap and thus only UV responsive. The second-generation photocatalysts (WO_3_, Fe_2_O_3_, and Cu_2_O) have the challenge of low quantum yield due to their rapid electron–hole recombination and poor stability (Kong et al., 2019). Perovskite and perovskite-related materials are considered as third-generation photocatalysts which form a stable structure and solid solution with several ranges of metal ions to achieve the appropriate band engineering for photoelectrocatalytic applications (Yang et al., 2009; Kong et al., 2019). Emphasis will be on photocatalysis and electrochemically advanced oxidation systems such as electrocatalysis and photoelectrocatalysis, with perovskites as the semiconductor. Prior to the discussions of the applications of the perovskites in water treatment, brief accounts on synthesis and types of this interesting material are provided.

## General Synthesis Methods for Perovskites

Perovskites are usually formed at elevated temperature because from their composition, perovskite oxides are compounds consisting of two or more simple oxides having high melting points (Arandiyan et al., 2018). The approach to synthesize perovskite oxide must be selected according to the specific application, specific demands of activity, and selectivity as these depend on the arrangement of atoms within its surface (Lim et al., 2019). For example, the solid-state synthesis method is commonly used to prepare perovskite in the pure form due to the availability of impurity-free precursors, and they find application in electronics. The downside of the solid-state synthesis approach is that it requires annealing at high temperature for a long time and frequent intermediary grindings which results in poor homogeneity as well as difficulty in controlling the particle size. Thus, problem arises when perovskites from solid-state methods are subjected to surface-related studies.

Since this review focuses on photocatalysis and photoelectrocatalysis, the methods discussed are those related to improved porosity, to achieve high surface area, *etc*. Efforts have been made by researchers in synthesizing perovskites at low temperatures with improved porosity (Labhasetwar et al., 2015). To overcome the homogeneity drawback of solid-state methods and to achieve nanocrystalline phase, reproducibility, and pure powder, several groups have focused on wet chemistry methods such as citrate sol–gel method, precipitation method, electrospinning technique, ultrasonic method, hydrothermal method, and microwave-assisted synthesis methods. Wet chemistry methods are characterized by their simplicity, reduced sintering time, mass production, high level of repeatability, lower temperature (than solid-state reaction), better flexibility in thin film–forming superior homogeneity, improved control of stoichiometry, purity, particle size, and a low industrialization implementation cost (Assirey, 2019). One of the challenges faced in the development of perovskite catalyst is obtaining the right structure and maintaining high surface area because of high calcination temperature employed sometimes during synthesis (Akinlolu et al., 2019). Hence, the choice of method of preparation is a top priority.

### Hydrothermal Method

The hydrothermal method is a useful technique for synthesizing perovskites. This method depends on solubility of minerals in hot water under high pressure, and many syntheses of perovskites for catalytic purpose have been carried out with various advancements using this method. The hydrothermal method is useful in perovskite synthesis as the particle size and shape can be affected by controlling the reaction temperature, pH, time, and concentration of reactants. Biasotto et al. (2011) hydrothermally synthesized bismuth ferrite (BFO) nanoparticles at a low temperature of 180°C within 1 h. In comparison with solid-state reaction process, the authors recorded submicron crystallites of BFO with enhanced homogeneity. Met and group (Kostyukhin et al., 2019) synthesized LaFeO_3_ *via* a hydrothermal microwave-assisted synthesis at a relatively low temperature of 240°C and pressure of 60 bar. In the procedure, the precursors were mixed in deionized water with the addition of KOH gradually while stirring. The presence of microwave as the heating source assisted in an enhanced crystallization rate of nanoparticles. Gao et al. (2015) synthesized BiFeO_3_ using nitrates of bismuth and iron *via* a hydrothermal method. KOH was added as a mineralizer to assist in the coprecipitation of Bi^3+^ and Fe^3+^. The XRD result shows that a single-phase cube-like BiFeO_3_ was successfully synthesized. Also, the effects of reaction time, KOH concentration, and organic dispersant on the BiFeO_3_ particle morphology size were investigated. The prepared photocatalyst showed excellent photodegradation of methyl orange dye (MO) under visible light (>420 nm).

Additionally, hydrothermal technique has also been adopted for the preparation of doped perovskites as well as perovskite-based heterojunctions. For instance, Baeissa, 2016 synthesized gold-doped NaNbO_3_ through a hydrothermal method for photocatalytic degradation of malachite green dye. The morphology of sodium niobate was studied by changing the hydrothermal temperature from 100 to 250°C. The XRD results reveal that all samples prepared at different temperature were of the perovskite structure. The hydrothermal temperature played an important role in the structure and surface area of the obtained sodium niobate as the temperatures at 100, 150, 200, and 250°C led to photocatalysts with surface area of 7, 9, 13, and 16 m^2^/g, respectively. Wen et al. (2017) synthesized a novel SrTiO_3_/BiOI heterostructure photocatalyst through hydrothermal and facile chemical bath methods with the aid of ethylene glycol. The XRD pattern of SrTiO_3_ depicts a characteristic tetragonal structure with a cubic SrTiO_3_ perovskite structure, and the morphology of SrTiO_3_ possesses nanosheets of irregular edges. It was also noticed that the SrTiO_3_ nanosheets fasten on the surface of BiOI nanoplates, forming a SrTiO_3_/BiOI. Photocatalytic activity, photoluminescence, and electrochemical impedance spectroscopy analysis confirmed higher photo-induced charge separation efficiency possessed in SrTiO_3_/BiOI composites, which they attributed to an intimate contact between SrTiO_3_ and BiOI.

**FIGURE 2:**
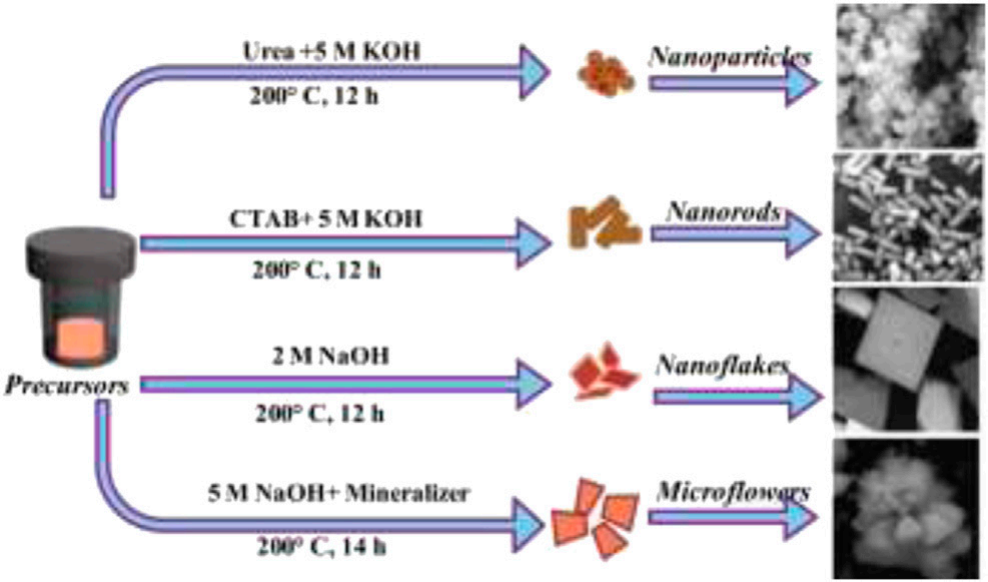
Schematic illustration for tuning the morphology BFO nanostructures with different experimental conditions by the hydrothermal process (Remya et al., 2020).

These reports show that hydrothermal synthesis is ideal for the preparation of pristine perovskites as well as composites containing perovskites. However, the efficiency of the hydrothermal method is dependent on various modifications of experimental conditions such as temperature and solvents used in dissolution of precursor reagents. Remya and group (Remya et al., 2020) synthesized BiFeO_3_ *via* hydrothermal methods with different experimental conditions for the fine-tuning of the final product (Figure 2).

### Citrate Sol–Gel Method

The citrate sol–gel method is usually used to prepare nanosized materials. Although its application is limited due to stability of its precursor system, it is difficult to control the chemical composition of complex oxides. The sol–gel procedure in aqueous medium uses inorganic salts and a chelating agent of carboxylic acid such as citric acid as a precursor. This technique has widely been used in making thin films with low temperature. Chu et al. (2018) reported a B site–deficient perovskite prepared *via* the classic sol–gel calcination method. In these methods, the nitrates of the metal ions were dissolved in deionized water, citric acid, and ethylene glycol to form a homogeneous solution at a certain pH, calcination temperature, and time. The XRD pattern showed that pure perovskite was successfully formed. SEM images revealed that most of the nanoparticles are non-agglomerated due to low calcination temperature. The prepared photocatalyst displayed a good photocatalytic property.

**FIGURE 3:**
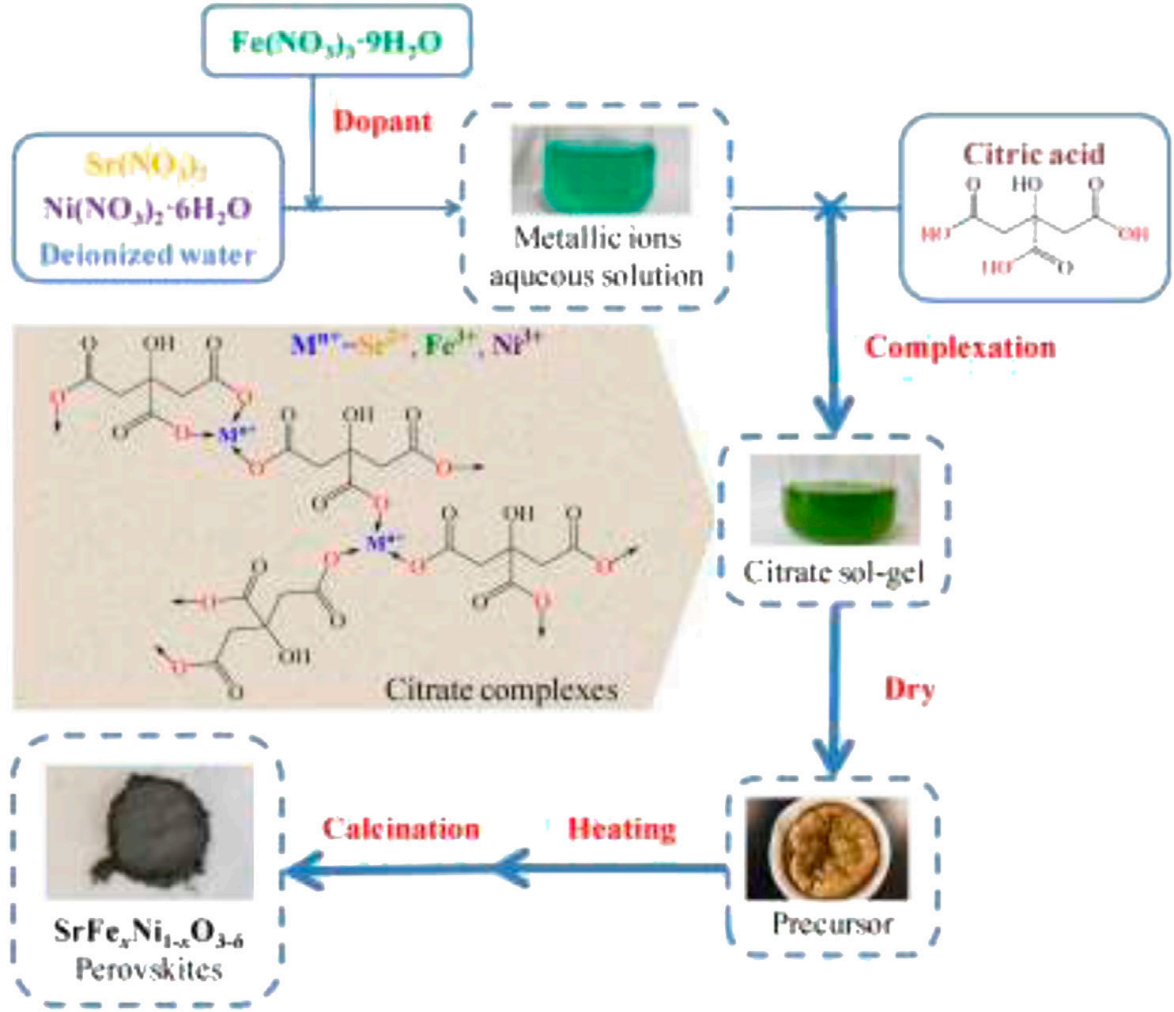
Schematic diagram of the fabrication process of the SrFe_x_Ni_1-x_O_3_-_δ_ perovskites (Zhang Y. et al., 2019).

In another report, LaMg_x_Fe_x-1_O_3-δ_ perovskite prepared *via* sol–gel route showed a formation of well-crystallized perovskite. LaMgFe_3_ and LaMgFe_4_ photocatalysts presented a particle size of around 100–150 nm with a well-defined size. The photocatalytic efficiency of LaMgFe_4_ was higher than that of other prepared catalysts owing to its smaller particle size distribution and higher surface area (Teresita et al., 2016). Zhang Y. et al. (2019) prepared SrFe_x_Ni_1x_O_3_-_δ_ (*x* = 0, 0.1, 0.2, 0.3, 0.4, and 0.5) *via* the citrate sol–gel method (Figure 3). They recorded their optimum catalyst with good electrocatalytic performance for water treatment was prepared at a reaction time of 120 min, a calcination temperature of 700°C, and Fe-doping content of *x* = 0.3.

### Coprecipitation Method

Coprecipitation occurs as a result of different cations in solution precipitating simultaneously, and this method encourages homogeneity of products. Coprecipitation plays a critical role in controlling the temperature, concentration, pH, and solution homogeneity. Haron et al. (2017) prepared a nanostructured perovskite oxide such as LaMO_3_ (M = Al, Co, or Fe) by the coprecipitation method. They observed a formation of single-phase nanocrystalline with high purity, larger surface areas, and porosity.

Djoudi reported a study on LaAl_1-x_Ni_x_O_3-δ_ prepared *via* the coprecipitation method using NaOH. They observed an increase in peak shift as the concentration of doping material increases, and crystallinity of all samples varies as the calcination temperature was adjusted. Therefore, the pure perovskite phase sample was calcined at 700°C for 6 h with no impurity. The morphology of the sample showed partial agglomeration, and interestingly, the material exhibited great electroactivity properties, which indicates a good electrocatalyst for oxygen reduction and evolution (Djoudi and Omari, 2015).

## Perovskite Oxide–Based Materials in Photocatalysis and Photoelectrocatalysis

### Perovskite Materials in Photocatalysis

In recent years, advanced oxidation processes (AOPs) have emerged to be efficient and effective methods for the treatment of wastewaters. They are utilized for the removal of organic pollutants during water treatment due to the generation and use of hydroxyl radicals as oxidizing species, which initiate other reactions for the degradation and possibly mineralization of organic pollutants. Among the AOPs, photocatalysis has attracted attention as a promising technique for solving environmental problems. Photocatalysis is a process that occurs when a semiconductor absorbs a photon of energy greater than its bandgap (the region between the electron-fill valance band and the empty conduction band of a semiconductor), and an electron is excited to the conduction band, thereby creating a hole in the valence band. The generation of the electrons could lead to oxidation and reduction reactions on the surface of the semiconductor (Umukoro et al., 2016). Semiconductors such as TiO_2_, SnO, and ZnO are widely used as a catalyst in photocatalytic reactions. TiO_2_ has been widely applied in photocatalysis owing to its stability, low toxicity, low cost, and high oxidation efficiency. However, its rapid recombination of photogenerated electron–hole pairs and low absorption of visible light are shortcomings that necessitate the need for other materials that have narrower bandgap and wider range of wavelength of light absorption in the visible range, and high solar energy. A suitable photocatalyst should have the following characteristics: i) it should have a bandgap ≥1.2 eV to provide energetic electrons and a bandgap ≤3.0 eV to allow effective absorption of overlap with the solar spectrum; ii) its photogenerated charge carriers should be easily available for use with the electrolyte, and it should be resistant to photocorrosion (Ge et al., 2016; Kumar A. et al., 2020). Other beneficial factors include low cost, facile preparation method, and amenability to bandgap tuning. Perovskites fit into these characterizes to a large extent. The shortcomings of recombination and photocorrosion can be minimized by perovskite composites such as in doping and/or heterojunction formation. Table 1 shows some recent perovskites that found their application on photocatalysis for water treatment. The different classes of perovskites used in PC are discussed in the following sections.

**TABLE 1:** Recent perovskites in photocatalysis for water treatment.

Perovskite	Co-catalyst	Light source	Method	Pollutant	Pollutant conc	Catalyst conc	Time	% Removal	References
BiFeO_3_	N-rGO	Hg arc lamp	—	Rhodamine B	100 mg/L	10 mg/L	3 h	98.7	Dixit et al. (2020)
LaTiO_3_	Ag	300 W Xe	Hydrothermal	Atrazine	—	1.2 g/L	40 min	100	Shawky et al. (2020)
SrTiO_3_	rGO	450 W Xe	Hydrothermal	Rhodamine B	0.04 mg	0.01 g	—	94.5	Rosy and Kalpana (2018)
LaMnO_3_	Ca	25 mW cm-2	Hydrothermal	Methylene blue	7 ppm	0.07 g/L	360 min	73	Arabi et al. (2018)
CaSnO_3_	rGO	UV	Microwave irradiation	Methylene blue	100 mg/L	0.1 g	150 min	92	Venkatesh et al. (2020b)
La_2_MnTiO_6_	—	400 W	Sol gel	AB 113	50 mg/L	30 mg/L	120 min	72	Shirazi et al. (2020)
SmFeO_3_	CuO	300 W	Sol gel calcination	Rhodamine B phenol	20 mg/L 20 mg/L	0.15 g	120 min	93 85	Behzadifard et al. (2018)
BiFeO_3_	BiOI	—	Hydrothermal	—	60 ppm	0.12 g/L	136 min	—	Bahmani et al. (2020)
SrTiO_3_	Ag/Ag_3_PO_4_	500 W Xe	Hydrothermal	Tetracycline	10 mg	0.2 g/L	15 min	72	Yu et al. (2020)
SrTiO_3_	La, Fe	150 W	Ball milling	Methyl orange	5 ppm	0.6 g/L	150 min	96	Abdi et al. (2020)
SrTiO_3_	La, Cr	300 W Xe	Sol gel hydrothermal	Tetracycline	20 mg/L	50 mg	90 min	83	Jiang et al. (2019)
LaNiO_3_	TiO_2_	300 W Xe	Sol gel	Methyl orange ciprofloxacin	10 mg/L 10 mg/L	100 mg 50 mg	150 min 210 min	100 54	Chen C. et al. (2020)
BiBaFeO_3_	Na, K	250 W Xe	Sol gel	Methylene blue	10 mg/L	15 mg	120 min	65	Haruna et al. (2020)
CaTiO_3_	2D/1Dg-C3N4	Sunlight	Hydrothermal	Crystal violet malachite	10 mg/L 10 mg/L	20 mg/L	180 min 90 min	99.76 95.02	Chen X. et al. (2020)
LaCoO_3_	Mn, N	32 W	Pechini-type sol gel	Malachite green	10 mg/L	40 mg/L	5.5 h	80	Nakhostin Panahi et al. (2020)
LaCoO_3_	Ag	—	Hydrothermal	Methylene blue	N/A	N/A	10 min	99	Jayapandi et al. (2018)
(NaBi) TiO_2_–BaTiO	Ag, N-Ni	300 W	Solid state	Rhodamine B	10 mg/L	1 g/L	80 min	92.4	Xiao et al. (2020)
CsPbBr_3_	QD	300 W	Precipitation	Tetracycline methyl orange	10 mg/L	100 mg	30 min	76 70	Qian et al. (2020)
SrZrO_3_	Sb_2_O_3_	450 W	Solid state	Tetracycline	10 ppm	0.2	180 min	70	Huerta-Flores et al. (2018)
[BaNiNbO_3_]	[KNbO_3_]	UV	Solid state	Methylene blue	20 mg/L	150 mg	120 min	55	Zhang et al. (2020)
CaTiO_3_	Bi_12_O_17_Cl_2_	300 W Xe	Hydrothermal	Tetracycline	0.02 mol/L	50 mg	30 min	90.7	Jiang et al. (2020)
LaFeO_3_	CA	100 W	Gel combustion	Azo dye RB5	30 mg/L	0.1 g	80 min	100	Yahya et al. (2019)
La_2_Ti_2_O_7_	CTAB	20 W	Sol gel	Azophloxine	40 mg/L	600 mg	180 min	—	Zhang et al. (2019)
AgLaTi_2_O6	Na, Cu	25 W	Sol gel	Congo red IWW	10–4, 50 ml	50 mg	60 min 240 min	24	Gade et al. (2018)
LaMnO_3_	—	Solar	Citrate sol gel	Methyl orange	6.5 ppm	N/A	60 min	100	Rekavandi et al. (2019)
CaTi0_5_	G	11 W UV	Solvothermal	Methyl orange	1 × 10–4 M, 50 ml	10 mg	36 h	N/A	Dong et al. (2020)
KTaO_3_	N	150 W	Solvothermal	Methylene blue	1 × 10-5 M	20 mg	360 min	Complete degradation	Rao et al. (2018)
MgTiO_3_	MgFe_2_O_4_	UV	Sol gel	Acid black	500 ppm	0.1 g	30 min	67.9	Kiani et al. (2019)
CaTiO_3_	Li, Ce	UV	Sol gel	Methyl orange	14 ppm	26 mg	150 min	90	Mužina et al. (2020)
BiFeO_3_	LNR	Sunlight	Sol gel	Methyl orange	10 ppm	0.15 g	3 h	98.9	Ridzuan et al. (2020)
SrSnO_3_	ZrO_2_	UV	Pechini method	Azo dye	10 mg/L	60 mg	10 h	98	Honorio et al. (2020)
La_2_Ti_2_O_7_	HTAB	UV	Sol gel	Olfloxacin	40 mg/L	30 mg	30 min	58.6	Han et al. (2020)
BiFeO_3_	—	100 W	Chemical route	Methylene blue	10 mg/L	20 mg	N/A	N/A	Verma et al. (2020)
LaFeO_3_	Au, Cu_2_O	200 W Xe	Sol gel	Rhodamine	5 mg/L	1 g/L	180 min	88.4	Guan et al. (2020)
BiFeO_3_	La	500 W	Sol gel	Methylene blue	15 mg/L	1 g/L	2.20 h	78.8	Kumar A. et al. (2020)
LaMnO_3_	CTAB	—	Hydrothermal	Methylene violet	15 ppm	5 mg	315 min	95	Priyatharshni et al. (2020)

#### Lanthanum-Based Perovskites in Photocatalysis

Lanthanum-based perovskites have raised interest as a result of their intriguing properties such as oxygen mobility, ionic conductivity, and excellent magnetic properties. For example, LaFeO_3_ had been proposed to be a significant material in photocatalytic areas because it exhibited several attention due to its properties such as narrow bandgap (E_g_ = 1.86–2.36 eV), more stability, and also environmental friendliness (Yahya et al., 2019). Thus, various synthesis approaches such as using template reagent and doping with other metals have been used to improve surface areas and enhance the charge transfer and light captivation through local surface plasmon resonance to improve its catalytic performance. For instance, Wang and group (Zhang W. et al., 2019) synthesized porous lanthanum–titanium La_2_Ti_2_O_7_ using CTAB as a template reagent through the sol–gel method toward the degradation of azophloxine dye. The authors observed that the presence of CTAB influenced the specific surface area and the crystallinity of the La_2_Ti_2_O_7_. The maximum number of hydroxyl radical was generated on the La_2_Ti_2_O_7_ sample obtained with 4 g of CTAB, which degraded about 100% of the dye after 180 min from UV-Vis absorption analysis. The photocatalytic degradation efficiencies for the first and the fifth cycles are 100 and 91%, respectively. The authors attributed this slight decline to the loss of fine La_2_Ti_2_O_7_ powder when taking solution samples for examination. This observation is consistent with one of the major challenges of photocatalysis–catalyst recovery. Also, in order to improve the photocatalytic performance of lanthanum-based perovskites, multiple complex or layered perovskite oxides containing lanthanum have been used. For example, Verduzco et al. (2018) synthesized a layered perovskite oxide Sr_2.7-x_Ca_x_Ln_0.3_Fe_2_O_7-δ_ (Ln = Nd and La) by a conventional solid–state reaction for degradation of methylene blue (MB) dye under solar and UV irradiation.

Varying the stoichiometry or doping of perovskite with a cation of different valence states can change the electronic structure, which dictates the electrical and optical properties (Zhang et al., 2010). The photocatalytic properties of Sr_3.2-x_Ca _x_La_0.8_Fe_1.5_Co_1.5_O_10-δ_ (Oliva et al., 2017) were studied by monitoring the degradation of methylene blue (MB) dye. This Ca-doped layered perovskite belonging to Ruddlesden–Popper (R-P) family exhibited increase in absorbance due to the creation of defects such as atomic dislocation induced by the replacement of Sr by Ca. It is worthy to note that the formation of these defects such as oxygen vacancies or trapping centers is suitable for photocatalysis because these oxygen vacancies diminish the rate of electron–hole recombination (Wu et al., 2016). As reported by Oliva et al. (2017), higher photocatalytic degradation was observed as the Ca content increased. The layered perovskites (SCLFCO sample) with *x* = 0.8 produced a total degradation of MB (100%) after 150 min at a pH = 6.0, whereas the undoped sample with *x* = 0 showed only a maximum degradation of 27% after 300 min. The authors attributed this to the lower bandgap, the low level of agglomeration, and particle size. The authors also explained that the chemical reaction for the degradation was due to the presence of Cl^−^ ion in MB molecule that balanced the charge with methyl group attached to a positive N^+^. So the presence of Ca^2+^ ion on the surface of the doped SCLFCO attracted Cl^−^ in the MB, thereby creating an unbalanced charge which facilitated the breaking of chemical bonds of the methyl groups attached to the chain of the MB molecule. Yang’s group (Guan et al., 2020) immobilized LaFeO_3_ and Au nanoparticles on the Cu_2_O surface as a ternary composite photocatalyst for rhodamine B degradation. The composite demonstrated a far more photocatalytic degradation performance than bare LaFeO_3_ and Cu_2_O. This avenue assists in facilitating the spatial separation of photo-induced electron hole. In another study, Ag nanoparticle was used to decorate LaTiO_3_ for the degradation of pesticide in water. The authors (Shawky et al., 2020) observed an improvement in the surface texture as observed from the morphological analysis. The composite demonstrated an enhancement in light absorption and reduced recombination rate as compared to the pure LaTiO_3_; as a result, complete degradation of atrazine pollutant within 40 min of irradiation was observed. Numerous reports have shown that incorporation or decoration of lanthanum-based perovskites with other metals or semiconductors results in pronounced enhancement in the photocatalysis (Arabi et al., 2018; Yahya et al., 2019; Chen C. et al., 2020; Nakhostin Panahi et al., 2020).

#### Non–Lanthanum-Based Perovskites

The applications of other non–lanthanum-based perovskites in photocatalysis have been reported. The following sections discuss ferrite-based, titanate-based, tantalum, and other types of perovskites for photocatalysis.

##### Ferrite-Based Perovskite in Photocatalysis

Iron-based perovskites have the general formula AFeO_3_, where A is a metal ion like Ca, Sr, Ba, Bi, La, Gd, Ga, or Y (Tang et al., 2011; Dhanasekaran and Gupta, 2012; Ramadan et al., 2013). Iron-based compounds could also adopt the FeAO_3_ structure, where A could be Ti (Gross-Sorokin et al., 2006; AlSalka et al., 2019). In this case, physical properties such as magnetism and/or ferroelectricity, which can be beneficial for the photocatalytic activity, are added to the material. Magnetism and/or ferroelectricity facilitate in extracting the photocatalyst from solution by an external magnet and also assist in the separation of the photogenerated charges.

Ferrite-based perovskites have proven to be promising materials for photocatalytic and photoelectrocatalytic applications in the field of environmental remediation. The interest and advantage of ferrite oxide could be attributed to its excellent properties such as narrow bandgap, nontoxicity, abundance of constituent element, low cost, and excellent electrical and catalytic properties (Liu et al., 2016). Ferrite-based perovskites attract attention due to their exceptional magnetic and electronic properties. They have intrinsic electric dipole moment due to a distortion in their crystal structures, which promotes separation of photogenerated charges during the photoexcitation process (Chen et al., 2017; AlSalka et al., 2019).

Most of the iron-based perovskites exhibit bandgaps within the visible region of the solar spectrum. For instance, bismuth iron oxide BiFeO_3_ (BFO), a multi-ferroic member of the iron-based perovskites, is a typical case where simultaneously and spontaneously antiferromagnetic (TN = 643 K) and ferroelectric (TC∼1123 K) order coexist well above room temperature (Sosnowska et al., 1992; Sati et al., 2015). BiFeO_3_ (BFO) with a rhombohedral distorted perovskite is a promising visible light responsive photocatalyst for organic pollutants degradation due to the suitable narrow bandgap (2.2–2.8 eV), excellent chemical stability, as well as intrinsic electric polarization (Ramadan et al., 2013). Charge-transfer (CT) transitions and spectroscopic measurements of the dielectric function of BiFeO_3_ single crystal (Pisarev et al., 2009) showed a defect-free intrinsic bandgap of ∼3.0 eV superimposed on a weak absorption band at 2.5 eV. This result signified the effect of defects and oxygen vacancies on the bandgap, and the shifting of the optical properties into the visible region. Electronic structure investigations have established the strongly hybridized nature of the valence band (Neaton et al., 2005; Clark and Robertson, 2007). However, some researchers reported that the photocatalytic activity of BFO was not impressive (Wang S. et al., 2016; Dixit et al., 2020), and they reported some drawbacks from BFO such as poor carrier mobility and rapid recombination of photogenerated electron–hole pairs. Based on these challenges, several strategies have been in place to improve its photocatalytic activity such as metal ion doping (Wang L. et al., 2016), heterostructure construction (Wang et al., 2017), structural control (Meng et al., 2016), and cocatalyst loadings (Behzadifard et al., 2018).

It has been recognized that metal ion doping assists in producing electron–hole trapping site which would probably accelerate the separation and transfer of the excited electron–hole pairs during the photocatalytic reaction (Wang S. et al., 2016; Dixit et al., 2020). Doping can also introduce surface defects such as oxygen vacancies by charge compensation arising between the dopant and the parent cation (Wang L. et al., 2016; Wang S. et al., 2016). Wang (Wang et al., 2017) studied the effects of oxygen vacancies induced by zirconium doping in bismuth ferrite for catalysis. The morphology features investigated by SEM and TEM showed the Zr-doped BFO with a smaller particle size of 50–150 nm significantly reduced aggregation as compared to the pristine BFO. The incorporation of Zr into BFO was confirmed by XPS. They reported that the optical absorption of the Zr-doped samples slightly shifted toward the shorter wavelength as opposed to the pristine BFO which has a strong absorption both in UV and visible light regions. A better photocatalytic performance in degradation of methyl orange (MO) using 2% Zr–BFO sample compared to the pure BFO and other Zr percentage loading samples was reported (Wang et al., 2017). Meng et al. (2016) studied the effect of the influence of lanthanum doping on photocatalytic properties of BiFeO_3_ for phenol degradation. The catalyst was prepared by a one-step facile sol–gel method using citric acid as the chelating agent. The gel was calcined at 500°C for 2 h and then at 600°C for 1 h in a muffle furnace. From the XRD result, the BiFeO_3_ structured was formed with the rhombohedral phase along with Bi_25_FeO_40_ impurity. As the wt% loading of La doping increases, the characteristic peak of the impurity disappeared. The result inferred that appropriate amount of La^3+^ doping can suppress the generation of impurity. SEM result also showed that 15 wt% La-doped BiFeO_3_ assisted to decrease the catalyst particle size. The bandgap of the samples was studied using the Kubelka-Munk (K-M) formula. The authors suggested that La doping narrowed the bandgap.

Several studies have been done on ferrite-based perovskites, and majorities of them are in combination with other metals, nanomaterials, and other semiconductors with a purpose of extending light absorption range, retarding the electron hole recombination, and boosting the electron mobility toward efficient charge separation to achieve an excellent photocatalytic degradation of pollutants in water (Behzadifard et al., 2018; Malathi et al., 2018; Yahya et al., 2019; Bahmani et al., 2020; Dixit et al., 2020; Diyan et al., 2020; Haruna et al., 2020; Kumar P. et al., 2020).

##### Titanate-Based Perovskites in Photocatalysis

Titanate-based perovskite (MTiO_3_) as a material has been explored in several applications. According to Alammar et al. (2015), they are promising materials for photocatalytic processes because of their excellent resistance to photocorrosion and high thermal stability. Ternary titanate-based perovskites such as CaTiO_3_, BaTiO_3_, and SrTio_3_ are wide bandgap titanate perovskite semiconductors with interesting electronic, optical, and magnetic properties (Alammar et al., 2015).

The preparation of titanate-based perovskite *via* the solid-state method often contains agglomerated particles of different sizes, morphologies, and impurities. Therefore, other methods where size, shape, and purity can be improved are favored. Titanate-based perovskites have offered applications in the area of photocatalytic hydrogen production and hydrocarbon reforming (Hbaieb et al., 2017). According to Zhang et al. (2007), the perovskite structure consisting of oxygen octahedral, such as TiO_6_, seems to play an important role for active photocatalysts, and the band edge positions, width of the conduction band, bandgap, and migration of photogenerated charge carriers as well as the photocatalytic activities are closely related to the distortion and the connectivity of the MO_6_ (M = Ti^4+^, Nb^5+^, and Ta^5+^) octahedra in perovskite titanates, niobates, and tantalates.

Titanate-based perovskites are reported to be photoactive materials, but their performance is impeded sometimes because of their wide bandgaps. However, titanate-based perovskites (such as SrTiO_3_ perovskite) exhibit good photostability and are thus applied in H_2_ production, solar cells, *etc*. Alammar et al. (2015) investigated the application of MTiO_3_ (M = Ca, Sr, and Ba) on the degradation of methylene blue using UV light. They reported that nitrogen-doped SrTiO_3_ showed good photocatalytic properties under visible light irradiation due to the formation of new states in the bandgap, allowing absorption of visible light. Huang S.-T.et al. (2014) studied the photocatalytic activity of SrTiO_3_ (STO) synthesized by the autoclave hydrothermal method under the alkaline concentration and time. In photodegradation of the model pollutant-crystal violet (CV) dye, STO prepared with 3 M NaOH for 72 h at 130°C showed best photocatalytic performance with UV light. In tailoring the optical and photocatalytic properties of SrTiO_3_, Lu et al. (2017) successfully incorporated bismuth ferrite into it, forming a structure Sr_1-x_Bi_x_Ti_1-x_Fe_x_O_3_. The synergetic effect resulted in more substantial visible light absorption, narrow bandgap, and enhanced photocatalytic performance. This study shows the possibility of tuning the bandgap of titanate-based perovskites into the visible light region. Lakhera et al. (2018) synthesized a visible light–responsive titanate-based perovskite by preparing a composite of TiO_2_ and NiTiO_3_ for photocatalytic degradation and hydrogen production activity. The photocatalytic activity of the nanocomposite was investigated by the photodegradation of rhodamine B dyes and tetracycline. With different calcination temperatures, the samples behaved differently. The optimized sample NT@750C nanocomposite displayed the highest degradation activity for RhB with about 75% total degradation within 2 h and nearly 58% degradation of tetracycline in 2 h with visible light. The total organic carbon (TOC) removal for both pollutants was lower than the respective decolorization rate, which is an indication of intermediate formation. The reusability test results for RhB dye showed that the photocatalyst can be reused for up to three cycles consecutively without major lost in the photocatalytic activity. In another study, Kiani et al. (2019) reported that a facile method was used to prepare magnesium titanate with magnesium ferrite nanocomposite as a support to improve the photocatalytic activity in degrading azo dyes. As a result of the heterostructure formed, the holes migrated to the valence band of MgFe_2_O_4_, while the electrons in the conduction band (CB) of MgFe_2_O_4_ migrated to the CB of MgTiO_3_, enabling an efficient charge separation and low recombination rate, hence allowing room for more charges to participate in redox reaction. Therefore, the photodegradation efficiency of the nanocomposite MgTiO_3_–MgFe_2_O_4_ increased about 30% relative to MgTiO_3_. Combination power of microstructure and heterojunction techniques in a quest to remould the inherent wide bandgap and enhance its visible light absorption capacity and photocatalytic performance cannot be down played. A multi-shelled cube structure of the CaTiO_3_–Bi_12_O_17_Cl_2_ heterostructure was synthesized by Jiang et al. (2020) for degradation of tetracycline in water. They observed a greater percentage of tetracycline degradation within a short period with the multi-shelled hollow cube CaTiO_3_–Bi_12_O_17_Cl_2_ heterostructure. They attributed the achievement to the larger surface area exhibited by the composite which led to more active sites than those participated in the redox reaction. There was a reduction of bandgap from 3.51 to 2.61 eV, thereby enhancing the absorption and utilization of visible light irradiation. The photochemical properties of the prepared samples which include photocurrent response, electrochemical impedance spectroscopy (EIS), photoluminescence spectra (PL), and linear sweep voltammetry (LSV) showed that the heterostructured photocatalyst exhibited an excellent photostability, preferable electron–hole pair separation and longer lifetime, less charge migration resistance, and highest electric current densities.

Several researchers have reported titanate-based perovskite with heterostructure and co-doping. A majority of them have demonstrated an excellent photocatalytic performance as compared to their bare samples, and this is due to reduced bandgaps and efficient migration of charge pairs, hence reduced recombination rate which produces great catalytic activity within the redox reaction (Rosy and Kalpana, 2018; Jiang et al., 2019; Abdi et al., 2020; Chen X. et al., 2020; Dong et al., 2020; Han et al., 2020; Mužina et al., 2020; Tomar et al., 2020; Xiao et al., 2020; Yao et al., 2020; Yu et al., 2020). For instance, Abdi recorded 96% photodegradation of methyl orange within 150 min with Fe–La–doped SrTiO_3_ nanoparticle as compared to 5% from bare SrTiO_3_ (Abdi et al., 2020). In addition, these heterostructures contribute in generating more h^+^ and superoxide O_2_, which are crucial oxidizing species involved in the catalytic system.

##### Tantalum and Other Perovskite-Based Materials in Photocatalysis

Tantalum-based perovskites (MTaO_3_) have received attention as a new class of material. They have been explored greatly in the area of water splitting. Kudo and Kato have carried out several studies on tantalum-related materials (Kato and Kudo, 1998; Kato and Kudo, 2001; Kudo et al., 2000). Tantalum-based perovskites (MTaO_3_) have also found applications in photocatalysis. Li and Zang (2011) prepared La-doped NaTaO_3_ *via* the hydrothermal method for the degradation of salfranine T dye under UV irradiation. The NaTaO_3_: La nanocube structure showed higher photocatalytic activity than the undoped. Usually narrow bandgap indicates convenient excitation from the valence band to the conductor band. Therefore, the authors attributed the higher catalytic activity observed on the doped material to the narrow bandgap it exhibited.

In the quest to improve the photocatalytic activity in perovskite, anion doping has been found attractive in improving the photocatalytic activity under visible light. Liu et al. (2011) synthesized N-doped NaTaO_3-x_ N_x_ *via* the one-step hydrothermal method for the degradation of methyl orange. Doping with nonmetals has been shown to increase the catalytic activity of material as it narrows the bandgap and creates oxygen vacancies. The photocatalytic decolorization efficiency of N-doped NaTaO_3_ was enhanced more than that of undoped because the interstitial nitrogen moved to the local state below the conduction band, and therefore excitation to the CB from the local state was convenient as a result of the “add on shoulder” on the N-doped NaTaO_3_. They recorded complete decolorization within 14 h irradiation of sunlight at pH 4 and COD removal percentage of 95.21% under the same conditions.

N-doped potassium tantalate perovskite has also been used for the photocatalytic degradation of dye (Rao et al., 2018). The authors recorded reduction of bandgap and also extension of absorption of light from the UV region to the visible region which accelerates the photocatalytic activity. Modak and Ghosh (2014) successfully synthesized N (p-type) and F (n-type) doping in NaTaO_3_ under visible light. Co-doping with N and F resulted in the formation of charge compensation and the isoelectronic system on NaTaO_3_. There was a reduction in bandgaps and visible light active material as a result of doping with N and F, which improved the photocatalytic activity. Apart from doping, the formation of heterojunction has also been reported for visible light photocatalytic applications. WO_3_-wrapped NaTaO_3_ prepared through a facile hydrothermal method was used to degrade tetracycline (Qu et al., 2015). An optimum percentage of 60.88% was recorded by the sample NaTaO_3_/WO_3_ which the authors attributed to an effective separation of the photogenerated electron–hole as well as the expansion of the absorption edge to the visible region due to the heterojunction structure.

Some other types of perovskites that have found applications on photocatalysis are discussed below. Huerta-Flores et al. (2018) synthesized the SrZrO_3_–Sb_2_O_3_ heterostructure for photocatalytic degradation of pharmaceutical compound. The heterostructure was successfully constructed using the impregnation method and was confirmed by XRD and SEM analyses. Results showed that they exhibited a weaker photoluminescence PL activity than Sb_2_O_3_ and SrZrO_3_, which is an indication that there was adequate charge separation and mobility in the interface of SrZrO_3_–Sb_2_O_3_, hence a minimized recombination rate. The open circuit potential (OCP) was used to evaluate the stability of the samples. The heterostructure exhibited good photostability because the presence of SrZrO_3_ inhibited the oxidation of Sb_2_O_3_. The higher amount of electron accumulated in the conduction band in the heterostructure was evident because the change in the OCP negative value from dark to light was higher. It was also recorded that Sb_2_O_3_ induced a rapid electron transfer to the solution, which was effective in avoiding the charge carrier recombination, thereby increasing the use of electron–hole pairs in the redox reaction. Results from the Nyquist plot showed a smaller arc radius for Sb_2_O_3_ and SrZrO_3_–Sb_2_O_3_ than the large semi-circle from SrZrO_3_ material. The decrease in the arc radius indicates a faster interfacial charge transfer. The authors summarized that as a result of diminished electron–hole recombination in the SrZrO_3_–Sb_2_O_3_ heterostructure, there was an increased availability of electron–hole separation which led to enhanced photodegradation of tetracycline.

Alkaline earth perovskite stannates such as ZnSnO_3_, CaSnO_3_, SrSnO_3_, and BaSnO_3_ have been widely used as photocatalysts. These materials have been found to be interesting for a number of potential applications in industry, such as components of dielectric ceramics; multifunctional signal sensors to detect temperature, humidity, and gas; as negative electrode active materials for long-life energy storage applications, and in the fabrication of ceramic boundary-layer capacitors (Zhang et al., 2006; Zhang et al., 2007; Fang et al., 2009). SrSnO_3_ has been explored and also proven to be an effective photocatalyst owing to its powerful oxidation and reduction capabilities. However, it is associated with large bandgap and a high recombination rate of photogenerated electron–hole pairs. Therefore, the applications of its heterostructured composites have attracted attention in recent years. For instance, Venkatesh et al. (2020a) synthesized a heterostructure rGO–SrSnO_3_ nanocomposite *via* hydrothermal methods. The authors recorded 97% methylene blue degradation and 80.66% for pure SrSnO_3_. The optical property showed that a wide absorption band was identified at 220–270 nm for pure and rGO–SrSnO_3_ composite, respectively. They recorded a reduced bandgap after incorporating rGO from 4.2 to 3.75 eV. Reduced graphene oxide has been an excellent electron acceptor with quick electron transport kinetics, and by that, the support of SrSnO_3_ with rGO assisted in transferring the photo-induced electron to participate in the reaction, thereby leading to enhanced photocatalytic performance. Zhang et al. (2020) synthesized [KNbO_3_]_0.9_-[BaNi_0.5_Nb_0.5_O_3-δ_] perovskite *via* a solid phase reaction method for methylene blue degradation. KBNNO perovskite has a bandgap of 1.39 eV, which is much smaller than that of the parent perovskite KNO. Incorporation of Ni^2+^ and K^+^ encourages oxygen vacancies and charge compensation, respectively. The photocatalytic degradation efficiency of KBNNO was 55% in 120 min. They concluded that the catalyst loading and dye concentration played a crucial role in the photocatalytic efficiency. Due to the octahedral tilting in the crystalline network of SnO_6_, this distortion plays an important role in the migration of photogenerated charge carriers in SrSnO_3_, CaSnO_3_, and BaSnO_3_ for photocatalytic degradation of pollutants (Honorio et al., 2020). Honorio et al. (2020) synthesized SrSnO_3_ with ZrO_2_ as a supporting semiconductor for photocatalytic degradation of a textile azo dye pollutant. Honorio discovered from SEM analysis that SrSnO_3_ was uniformly dispersed on ZrO_2_ which infers higher activity as a result of SrSnO_3_ active phase even in a smaller amount. Therefore, they recorded 63 and 98% degradation at time 2 and 10 h, respectively. But, the doping of SrSnO_3_ with ZrO_2_ did not significantly affect their bandgaps. They attributed this to be the presence of Sn(ll) having lower binding energy may have occupied the inter-band states below the conduction band of Sn(IV). Some studies have been carried out on Tin-based perovskites for photocatalysis which have yielded excellent catalytic performance (Wang et al., 2009; Junploy et al., 2013; Wang et al., 2014).

### Photoelectrocatalysis

Photocatalysis has been widely utilized, and it is an important route for the degradation of organic waste. Its efficiency strongly relies on the catalyst. This technique has prominent advantages, including low cost and no secondary pollution. However, it has some drawbacks such as electron–hole pair recombination and catalyst recovery. Electrochemical oxidation localizes the semiconductor in the form of an electrode or substrate. Thus, the degradation of the organic pollutants occurs at the electrode surface or close to the electrode surface (hydroxyl radical in the bulk solution). This approach solves the catalyst recovery problem in photocatalysis. This electrochemical approach, however, requires high voltage and is prone to oxygen evolution, and the process is limited by mass transfer (Malpass et al., 2010; Sirés et al., 2014; Brillas and Martínez-Huitle, 2015; Kusmierek and Chrzescijanska, 2015; Umukoro et al., 2017; Wang et al., 2018). The quest for improved techniques led to the emergence of photoelectrochemical oxidation or photoelectrocatalytic (PEC) processes. PEC is an electrolytic system containing a semiconductor-based anode that is simultaneously subjected to light illumination and a constant bias potential to the anode (E_anod_), a constant cell potential (E_cell_), or a constant anodic current density (j_anod_). This process promotes the extraction of photo-induced e^−CB^ by the external electrical circuit, thereby yielding an efficient separation of the e^−CB/h+^_VB_ pairs (Daghrir et al., 2012; Georgieva et al., 2012; Sirés and Brillas, 2012; Bessegato et al., 2015; Meng et al., 2015). The prevention of charge recombination promotes the photocatalytic efficiency of the anode and thus the acceleration of organic oxidation. The excitation of the anodic semiconductor and prolonged separation increases the lifetime of the hole so as to have more opportunities to either directly oxidize the organic pollutants adsorbed on the photoanode surface or indirectly react with absorbed water to form more hydroxyl radicals. Another advantage of PEC is the ease of recovery of photocatalyst after usage and possibility of multiple cycles of treatment. A myriad of semiconductor materials such as TiO_2_, ZnO, WO_3_, and BiVO_4_ have been used as photoanodes in PEC. Another class of semiconductor used in PEC is perovskites. As seen in photocatalysis, their recent trend is to use visible light as the photoexcitation source for the anode.

#### Perovskite in Photoelectrocatalysis for Water Treatment

Perovskites have been extensively used in photocatalysis. In the area of photoelectrocatalysis (PEC), however, most applications of perovskites are in the energy sectors such as water splitting (Ni et al., 2007; Bin Adnan et al., 2018; Phoon et al., 2018) and solar cells (Muñoz-Gil et al., 2018; Fan et al., 2020). The application of perovskite in PEC for water treatment is still at its infancy. This review seeks to capture the recent application of perovskites in PEC and thus provide a valuable resource for materials, perovskites, and environmental research communities. Reports on PEC for water treatment are shown in Table 2 and reviewed in the following paragraphs. The chemistry of catalyst (perovskites) improvement strategies is similar to that of those explained earlier. Owing to the need to fabricate an anode (usually by depositing the semiconductor), different synthesis and electrode preparation methods peculiar to PEC are expected.

**TABLE 2:** Recent perovskites in photoelectrocatalysis for water treatment.

Perovskite	Cocatalyst	Light source	Method	Pollutant	Substrate	Pollutant conc	Catalyst conc	Time, min	% Removal	Ref
BiFeO_3_	Sm, Pd	300 W, Xe lamp	Conventional sol-gel	Methyl orange, phenol	FTO	5 mg/L	0.3 g	120	8750.1	Wang L. et al. (2016)
SrTiO_3_	TiO_2_	300 W, Xe lamp	Hydrothermal/anodization	Methylene blue	Ti	N/A	N/A	20	99.93	Huang J.-R. et al. (2014)
BiFeO_3_	TiO_2_-NT	500 W, Xe lamp	Citric sol-gel/ultrasonic immersion	Rhodamine	Ti	20 mg/L	N/A	150	100	Zhu A. et al. (2014)
LaFeO_3_	SrTiO_2_	420 nm LED	Ultrasonic spray pyrolysis	NO	FTO	400 ppb	0.1 g	N/A	40	Zhang et al. (2017)

**FIGURE 4:**
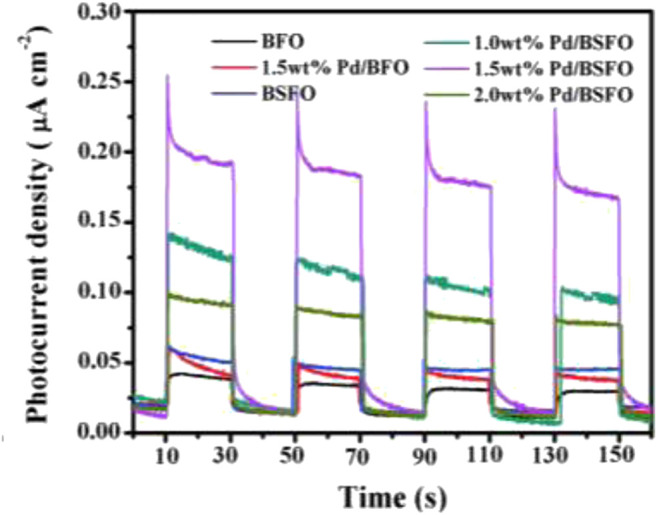
Photocurrent action spectra of the prepared photocatalyst samples (Wang S. et al., 2016).

The first task in the application of perovskites in photoelectrocatalytic removal of organic pollutants in water is to understand the electrochemical and photoelectrochemical behaviors of the perovskites. To get such information, the perovskites have to be fabricated into an electrode. One of the common substrates for the deposition of perovskites for PEC application is the fluorine-doped tin oxide glass (FTO). For example, Fermin and coworkers studied the photoelectrochemical properties of LaFeO_3_ nanoparticles by pasting an adhesive film of LaFeO_3_ (along with other diluents) on a FTO coated glass (Celorrio et al., 2014). Although this work was not on water treatment, valuable data such as photocurrent responses obtained from this work are necessary characterizations that inform the potential applicability of the perovskites for PEC water treatments. With the use of a similar electrode preparation strategy, Zhang carried out a robust study on the photoelectrochemical behavior of heterostructured perovskite of the LaFeO_3_–SrTiO_3_ composite on FTO glass for NO removal (Zhang et al., 2017). The electrode was characterized along with the control electrodes of pristine LaFeO_3_ (LFO) and SrTiO_3_ (STO). While all the photoanodes showed good photoelectric responses, the composite (LFO–STO) showed very pronounced response, indicating that heterojunction greatly promotes the separation of photo-induced electron–hole pairs and leads to the improved catalytic reaction rate. The authors concluded that the improved catalytic activity is due to the broad visible light harvest, enlarged surface area, and a suppressed surface charge recombination that emanated from the perfectly matched LaFeO_3_ and SrTiO_3_ interface and facile charge transfer. Wang S. et al. (2016) also carried out photoelectrochemical studies to investigate the effect of a Pd cocatalyst and samarium doping in bismuth ferrite (BFO). Accordingly, the authors recorded a higher photocurrent response in Pd/BSFO (BSFO = Sm-doped BFO) samples than in BFO, Pd/BFO, and BSFO. They concluded that photocatalytic analysis and electrochemical analysis such as photocurrent response as shown in Figure 4 demonstrated that the combination of Pd loading and Sm doping could significantly promote the separation of photogenerated electron–hole pairs in the Pd/BSFO sample, thereby improving the photocatalytic activities.

As demonstrated by Arotiba and coworkers, such a photocurrent response from a photoanode can give a measure of how effective the electrode will be when used for PEC degradation (Umukoro et al., 2018; Orimolade et al., 2019; Orimolade et al., 2020; Orimolade and Arotiba, 2020).

**FIGURE 5:**
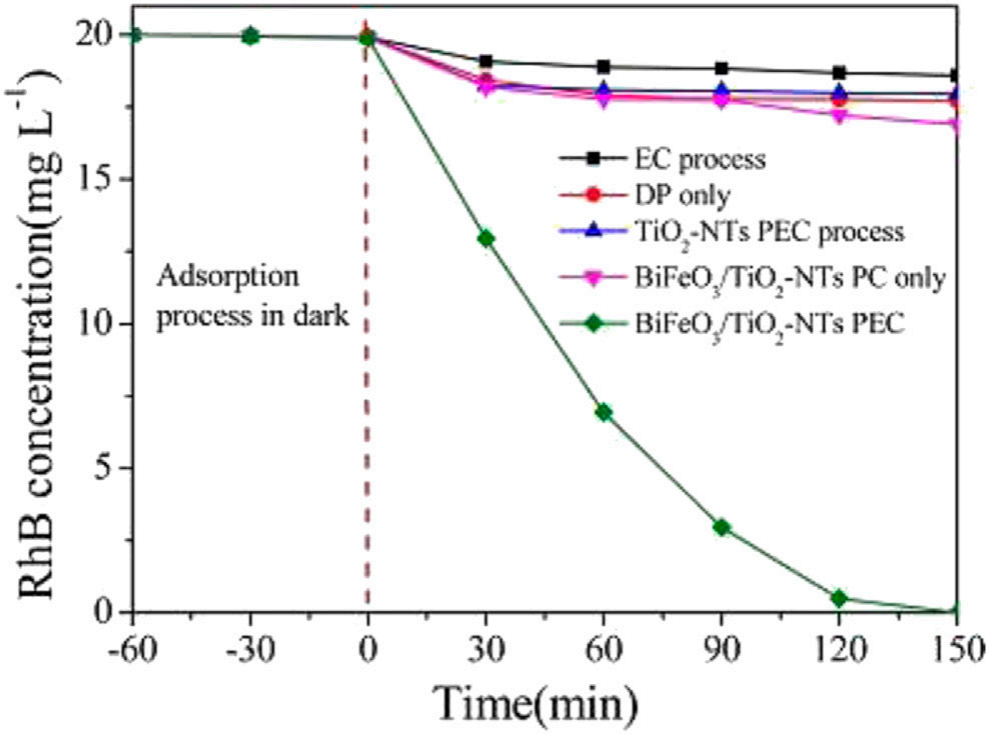
RhB concentration varying with time through different degradation processes. PC, photocatalytic process; EC, electrochemical oxidation. Note: label “b” is from the original author reproduced with permission (Zhu J. et al. (2014).

**FIGURE 6:**
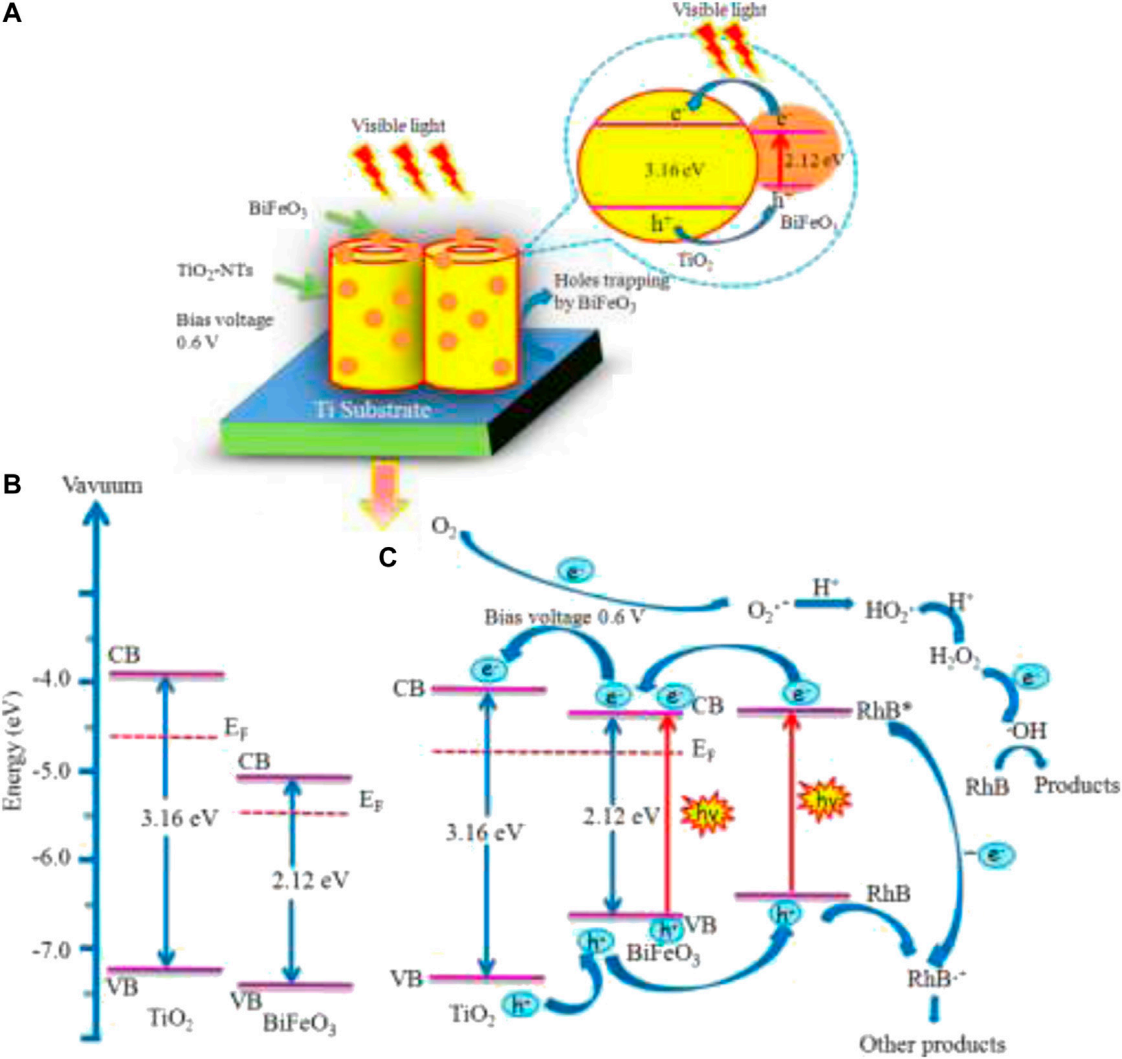
**(A)** Cartoon diagram of the photogenerated electron generation, separation, and transfer path in BiFeO_3_/TiO_2_-NTs upon visible-light excitation. **(B)** Conduction band, valence band, and Fermi-level positions of independent n-type TiO_2_ and n-type BiFeO_3_. **(C)** Schematic pathways of PEC degradation of RhB over BiFeO_3_/TiO_2_-NTs under a bias of 0.6 V applied onto the composite electrode (Zhu A. et al., 2014).

One of the earliest work where a perovskite based photoanode was prepared and used for the PEC degradation of an organic pollutant is the work of (Zhu A. et al., 2014). The authors prepared TiO_2_ nanotubes (TiO_2_-NT) by anodic oxidation of titanium sheet and then deposited the BiFeO_3_ nanoparticles from its precursor on the TiO_2_-NTs via an ultrasonic-immersion strategy to form the BiFeO_3_/TiO_2_-NTs photoanode. The electrodes were extensively characterized by UV−vis diffuse-reflectance spectra, surface photovoltage, photoluminescence, electrochemical impedance spectroscopy etc. As shown in Figure 5 with the applied bias potential of 0.6 V, the photoanode displayed a pronounced efficiency as compared to photocatalysis and electrochemical analysis. This is in agreement with the electrochemical impedance that the photoanode BiFeO_3_/TiO_2_-Nts possessed a faster charge transfer across the electrode surface to the Ti substrate. These results point to a marked improvement in the photo-electrochemical properties of the BiFeO_3_/TiO_2_-NTs over TiO_2_-NTs. These improvements were attributed to ease of charge transfer between the two materials, the ferroelectric properties of BiFeO_3_ and the enhancement of charge separation. The BiFeO_3_/TiO_2_-NTs was used in the degradation of rhodamine B dye. A degradation of nearly 100% was achieved at the BiFeO_3_/TiO_2_-NTs photoanode after 150 min. This was significantly higher than when other techniques such as electrochemical oxidation, direct photolysis and photocatalysis process were employed. The schemes suggested by the authors for the electrode, bandgap alignment and schematic pathways are presented in Figure 6. From the diagram, the formation of heterostructure assisted in the formation of appropriate Fermi level, the application of bias potential channelled the electrons generated from the conduction band of BiFeO_3_ to the conduction band of TiO_2_-NTs. The separated, photogenerated electrons reacted with the surface chemisorbed O_2_ to generate the strong oxidative species O^2−^, which combines with H^+^ from solution to form H_2_O_2_. Finally, accumulated electrons in the counter electrode can react with H_2_O_2_ to generate OH. This effective charge separation attributes to an excellent oxidation and reduction of the pollutants.

The positive coupling effect of combining TiO_2_ and other semiconductors is considered as an efficacious approach to promote TiO_2_ reactive properties. This enables the photogenerated charge carriers to migrate to the heterostructured interface to retard recombination. Huang J.-R.et al. (2014) constructed a heterostructured nanotube with TiO_2_/SrTiO_3_ for PEC degradation of methylene blue (MB) dye. The photocurrent response among all the prepared catalyst TSr3 (TNT in Sr(OH)_2_, 30 min hydrothermal time) showed the highest peak current, and it is twice as large as TNT2 (TiO_2_ nanotube on Ti substrate). Their photocurrent response is such that when light is turned off, they returned instantly to zero; hence, the author concluded that the heterostructure composites under UV light can significantly promote the photogenerated electron/hole separation. The PEC degradation peak value was in good agreement with the rate constant K with the optimal sample TSr3. The MB percentage degradation and rate constant were 99.93% and 0.38492 E^−4^ min^−1^, respectively. The authors attributed the result to be as a result of the positive cooperation of the high-reactive TiO_2_ {001} facet with TiO_2_/SrTiO_3_ heterostructured interface which strongly inhibited the photogenerated electron/hole recombination.

#### Effect of pH, Initial Concentration, and Catalyst Loadings of Perovskites in Photocatalysis and Photoelectrocatalysis

##### Effect of pH

The pH of a solution influences the surface charge and protonation of the functional groups in a catalyst. It also promotes the efficiency and the performance of the process. Haruna et al. (2020) studied the pH of the solution for the degradation of 10 mg/L methylene blue using Bi_0.85-x_M_x_Ba_0.15_FeO_3_ (M = Na, K and x = 0, 0.1) at pH 3, pH 7, and pH 11. The observed improved photodegradation at pH 7 and pH 11 of doped and undoped catalyst was attributed to the interaction of the highly negative oxide species and the anion which influenced the surface charge of the photocatalyst. Another study shows the degradation of tetracycline at pH 3, pH 5.60, and pH 10 using tungsten-doped BaTiO_3_. The authors recorded the degradation rate of 90 and 80% for pH 10 and pH 5.60, respectively, after 3 h irradiation and 43% at pH 3. They concluded that increasing OH ions on catalyst surface promoted more OH^−^ radicals which participated in the redox reactions (Demircivi and Simsek, 2019). Depending on the nature of the pollutant and the photocatalyst, the degradation efficiency varies with pH of the solution, hence the need to detect the optimum conditions that are favorable at any given time. Simsek et al. (2020) studied the effect of pH in photocatalysis and photo-Fenton for the degradation of caffeine using LaFeO_3_. They observed that the photocatalyst performed optimally at natural pH 6.5 compared with pH 3 and pH 10. According to Yao, the effect of pH and degradation of pollutant varies, and it is controversial (Haruna et al., 2020; Yao et al., 2004).

#### Effect of Initial Concentration of Pollutants

The increase in pollutant concentration could decrease the penetration of light into the solution for photocatalytic activity, thereby causing turbidity and opaqueness. Yahya et al. (2018) investigated the effect of initial concentration of humic acid using LaFeO_3_. They observed the photocatalytic removal in the order of 98, 90, 85, and 86% for 10, 20, 30, and 40 g/L, respectively. It shows that at lowest initial concentration of humic acid, the photocatalytic activity was at its best. Therefore, they concluded that at a high pollutant dosage, the penetration of light needed to travel to the active site to activate the photodegradation was blocked, resulting in few active sites and poor photocatalytic activity. Also, a similar result was obtained by Pelin, where the degradation of tetracycline solution with an initial concentration of 5, 20, and 40 mg/L showed a degradation rate of 93, 80, and 47%, respectively (Demircivi and Simsek, 2019). Increase in turbidity, which can be due to high initial pollutant concentration is expected to reduce the efficiency of photoelectrocatalytic degradation of pollutants with perovskite-based photoanodes. This is because of insufficient light reaching the photoanode owing to poor penetration or scattering just as it is observed in photocatalysis. The issue of turbidity is not restricted to the pollutant concentration alone. Thus, for practicality, the process of simple filtration or sedimentation to remove suspended solids may precede PEC to improve the delivery of light onto the electrode or the catalyst.

#### Effect of Catalyst Loadings

Usually, at lower catalyst dosage, there are few active sites available for photodegradation, and at higher catalyst loadings, there are agglomeration, high turbidity, and scattering effects which lower the degradation efficiency in photocatalysis (Yahya et al., 2018; Yahya et al., 2020). Behzadifard et al. (2018) investigated the effect of CuO (10 wt%) SmFeO_3_ composites for degradation of 10 mg/L rhodamine dye. They reported that the degradation rate of rhodamine increased with increasing catalyst loading from 0.05 to 0.15 g; however, at 0.20 g, the performance dropped, which they attributed to agglomeration and decrease in light penetration. It is therefore crucial to obtain the optimum value of catalyst loadings for cost-effectiveness and better photocatalytic performance.

## Bandgap Positions and Charge Transfer Mechanism of Perovskite Oxides in Photocatalysis and Photoelectrocatalysis

**FIGURE 7:**
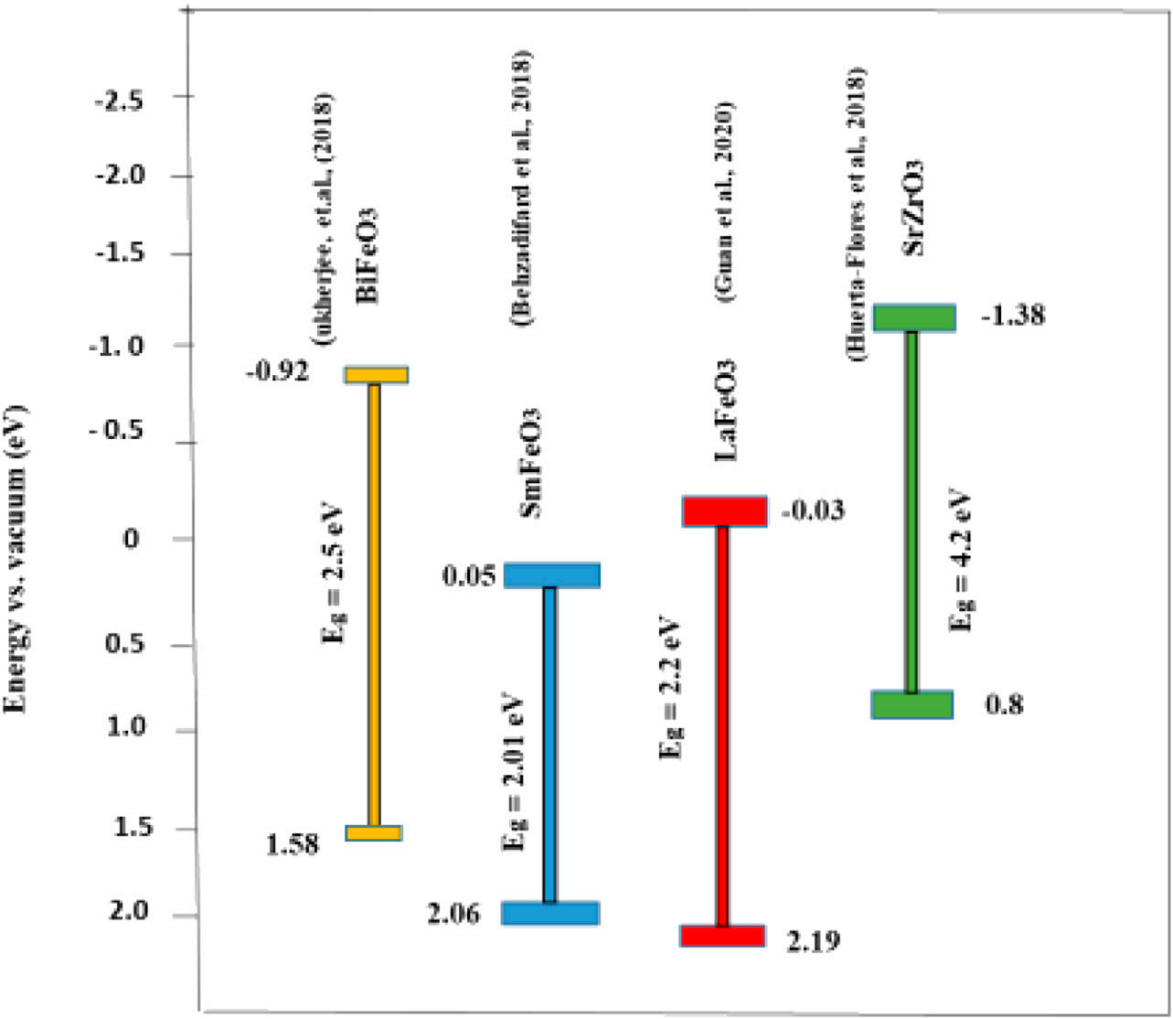
Band positions of some perovskite oxide–based materials in photocatalytic and photoelectrocatalytic treatment of water.

**FIGURE 8:**
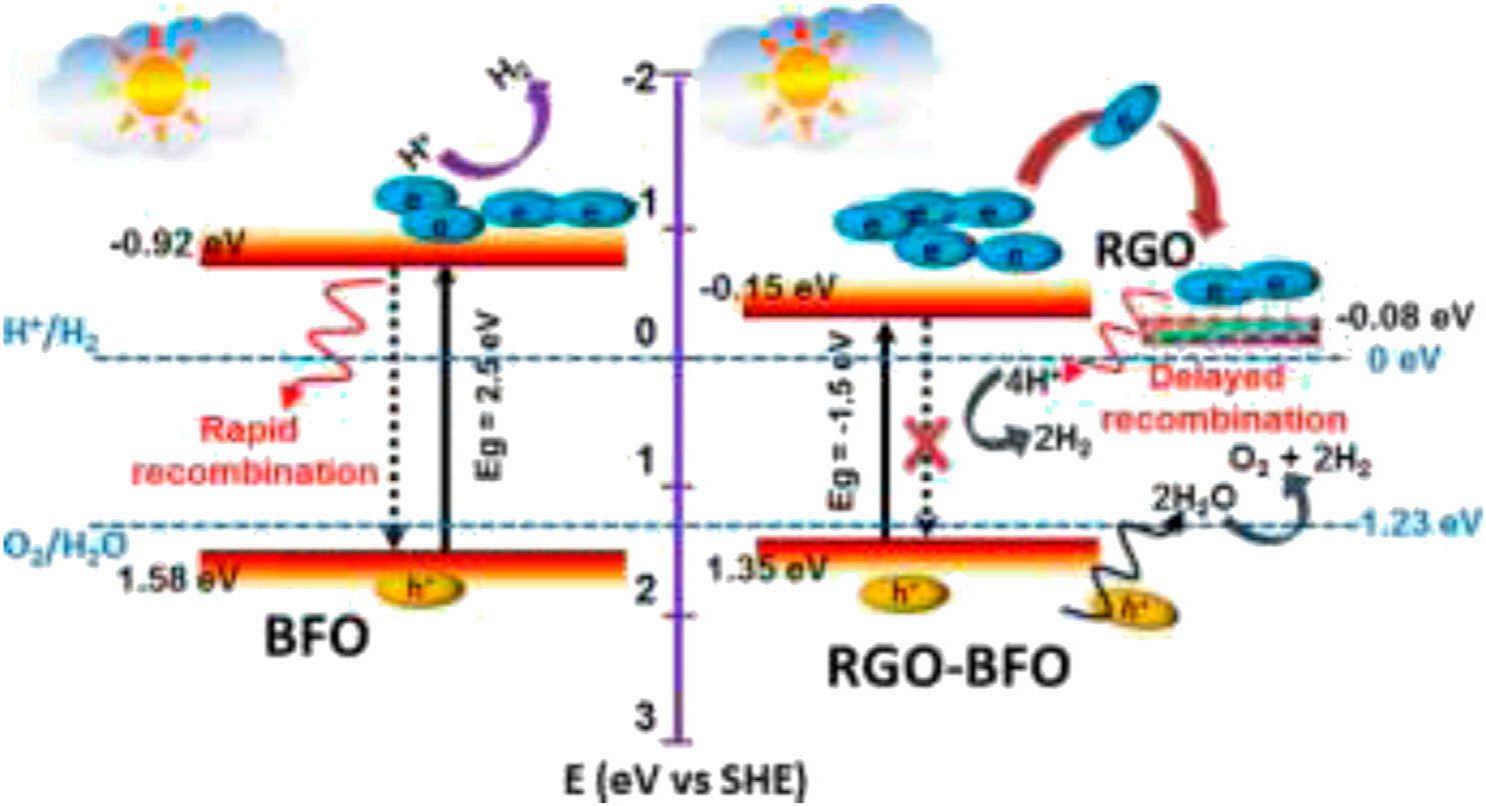
Schematic representation of the proposed mechanism of photocatalysis of BFO and RGO–BFO reproduced with permission (Mukherjee et al. (2018)).

**FIGURE 9:**
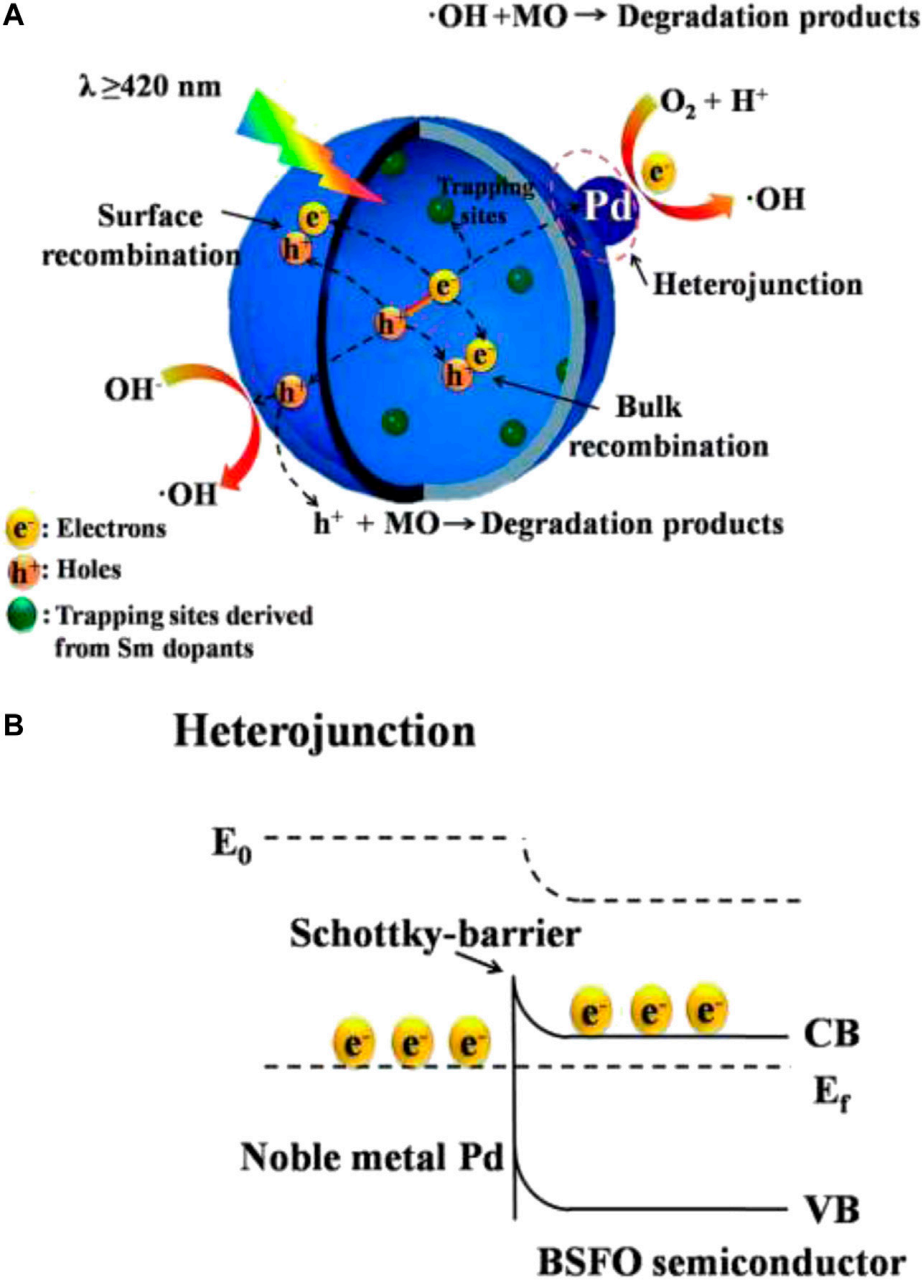
**(A)** Schematic diagram for the proposed visible light photocatalytic mechanism of Pd/BSFO samples and **(B)** schematic illustration of Schottky barrier formed at the interface between Pd and BSFO (reproduced with permission (Wang S. et al., (2016).

Bandgap calculation, tuning, and alignment are important in the characterization and in the prediction of the photocatalytic and photoelectrocatalytic efficiency of perovskite oxides (and other semiconductors) in water treatment. There are several ways of calculating band edge positions such as electronegativity-based calculation, density functional theory (DFT), the Mott–Schottky plot, dependence of photovoltage on pH, and photocurrent-potential measurement (Zhang and Jaroniec, 2018). Some band edge potentials of selected perovskite oxides discussed in this review are shown in Figure 7. Mukherjee et al. (2018) highlighted the effect of bandgap on photocatalytic improvement by the preparation of a composite of BFO and reduced graphene oxide (RGO). The conduction band (CB) and valence band (VB) edge potentials suggest band bending which arises in the equilibration of the Fermi level with the increase in the space region of the composite. The increase in the space region of the BFO–RGO composite resulted in the negative shift in the band edge potential, which facilitated the charge carrier concentration and consequently increased the chances of faster electron transfer, suppressed recombination, and better photoelectrocatalytic degradation (see Figure 8). In another report, Wang S. et al. (2016) explored the effect of rare earth (Sm) doping and noble (Pd) cocatalyst doping in BiFeO_3_. The flat band potential measurement was calculated using the Mott–Schokkty plot. Doping of bismuth ferrite with foreign atoms in their A, B, or O sites of the ABO_3_ lattice has been proven to be an effective route to improving its photocatalytic properties (Mukherjee et al., 2018). From the band diagram in Figure 9, as reported by Wang L. et al. (2016), the hole could react with H_2_O on the surface to generate OH for oxidation reaction because the VB potential of 1.5 wt% Pd–BSFO composite (+2.35 eV) is positive enough when compared to −OH/^**.**^OH (+2.27 V vs NHE) as opposed to the CB potential of 1.5 wt% Pd–BSFO (+0.27 eV) which is less negative than the potential E^o^ (O_2_/O_2_^**.**^) (−0.046 V vs NHE). Doping with rare earth metal served as an electron trapping specie to separate the photogenerated electron–hole from recombination, thereby promoting efficient charge carrier separation and consequently encouraging excellent photocatalytic activity. Behzadifard et al. (2018) studied the photocatalytic degradation of rhodamine and phenol. The band edge potentials were calculated using electronegativity methods. From the calculations, the VB and CB potentials for CuO were 1.97 and 0.65 eV and those of SmFeO_3_ were 2.06 and 0.05 eV, respectively. Owing to the low negative CB potential value of SmFeO_3_, adsorbed oxygen cannot be converted to −O_2_. On the other hand, the CB potential of CuO is more positive than SmFeO_3_; hence, electrons can migrate to the CB of CuO. In other words, CuO is acting as electro acceptor and is able to trap the electrons. Also, the CB potential of CuO (0.65 eV) is positive enough and closer to the potential for O_2_/H_2_O_2_ (+0.695 eV), and the electrons are able to react with adsorbed oxygen to produce H_2_O_2_ and subsequently ^**.**^ OH. The holes generated were transferred from the VB of SmFeO_3_ to CuO because the VB potential of SmFeO_3_ (+2.06) was not high enough to oxidize the adsorbed –OH (−OH/^**.**^OH = +2.38 eV) and H_2_O (H_2_O/^**.**^OH = +2.72 eV) to OH; hence, holes in SmFeO_3_ could have directly oxidized the organics had it enough potentials. The formation of the heterojunction promoted the charge separation and suppressed the recombination of charge carriers.

The mechanism of reaction was summarized in Eqs. 1–4 (Behzadifard et al., 2018; Zhang and Jaroniec, 2018).

$${\text{CuO/SmFeO}}_{3}+\text{hv }\left(\text{visible light}\right)\to {\text{e}}^{-}{\text{CB }(\text{CuO and SmFeO}}_{3}{)+\text{ h }+\text{VB}(\text{ CuO and SmFeO}}_{3}),$$ (1)

$${2\text{e}}^{-}\text{CB }\left(\text{CuO}\right)\;+\;2\text{H}+{\;+\text{ O}}_{2}{\text{ H}}_{2}{\text{O}}_{2},$$ (2)

H2O2+e-→O−H+OH, (3)

Pollutant + OH/h→+Intermediates→degradation products. (4)

## Conclusion and Recommendation

In this review, we discussed the recent progress in the field of photocatalysis and photoelectrocatalysis based on perovskite and perovskite-related materials. Recently, research has geared toward photocatalytic materials that are capable of absorbing light in the visible region. Perovskite and perovskite-related materials such as BiFeO_3_, LaMnO_3_, and LaFeO_3_, which are mostly visible–light active materials, are gaining attention as suitable anodic material for photoelectrocatalysis in degradation of organic pollutant due to their fascinating properties. Few photoelectrocatalysis degradation reports discussed here show that synergy of photocatalysis and electrolysis promotes better degradation performance. Reports have also shown that immobilizing catalyst onto substrate and application of bias potential aid in catalyst recovery, reduce electron–hole recombination, and increase charge resistance. In particular, the formation of perovskites as photoanodes through novel nanostructure engineering, surface modification with exotic element doping or cocatalyst loading, and innovative system design based on heterojunction configuration are excellent strategies for improving light harvest, charge separation, as well as surface reaction kinetics as compared to the single perovskites, for example, BiFeO_3_ doped with Sm and Pd (Wang S. et al., 2016). Agreeably, great progress has been made over the years; however, for ultimate realization of the inherent potential in perovskite, more work needs to be done in discovering new perovskites for photoelectrocatalysis. Furthermore, in order to maximize the degradation efficiencies in vast composition of organic pollutants, other advanced oxidation processes such as sono-photoelectrocatalysis and photo-Fenton process can be incorporated with perovskite material for complete mineralization of the recalcitrant pollutants.

Therefore, for the advancement of perovskite-based material for photoelectrocatalysis treatment of water, continuous research into a better crystal structure for stability as well as performance improvement is essential. Perovskites with long-term stability and exceptional optical and electrochemical properties are essential for sustainability.
